# Role of glycogenolysis in stimulation of ATP release from cultured mouse astrocytes by transmitters and high K^+^ concentrations

**DOI:** 10.1042/AN20130040

**Published:** 2014-01-13

**Authors:** Junnan Xu, Dan Song, Qiufang Bai, Lijun Zhou, Liping Cai, Leif Hertz, Liang Peng

**Affiliations:** *Department of Clinical Pharmacology, China Medical University, Shenyang, P. R. China; †Laboratory of Molecular Biology, Liaoning University of Traditional Chinese Medicine, Shenyang, P. R. China

**Keywords:** adenosine, astrocyte, ATP release, glutamate, glycogen, potassium, DAB, 1,4-dideoxy-1,4-imino-D-arabinitol, dBcAMP, dibutyryl cAMP, DMEM, Dulbecco’s Minimum Essential Medium, ERK, extracellular-signal-regulated kinase, GABA, γ-aminobutyric acid, MEK, [MAPK (mitogen-activated protein kinase)/ERK (extracellular-signal-regulated kinase)] kinase, MPEP, 2-methyl-6-(phenylethynyl)-pyridine, RLU, relative light unit(s)

## Abstract

This study investigates the role of glycogenolysis in stimulated release of ATP as a transmitter from astrocytes. Within the last 20 years our understanding of brain glycogenolysis has changed from it being a relatively uninteresting process to being a driving force for essential brain functions like production of transmitter glutamate and homoeostasis of potassium ions (K^+^) after their release from excited neurons. Simultaneously, the importance of astrocytic handling of adenosine, its phosphorylation to ATP and release of some astrocytic ATP, located in vesicles, as an important transmitter has also become to be realized. Among the procedures stimulating Ca^2+^-dependent release of vesicular ATP are exposure to such transmitters as glutamate and adenosine, which raise intra-astrocytic Ca^2+^ concentration, or increase of extracellular K^+^ to a depolarizing level that opens astrocytic L-channels for Ca^2+^ and thereby also increase intra-astrocytic Ca^2+^ concentration, a prerequisite for glycogenolysis. The present study has confirmed and quantitated stimulated ATP release from well differentiated astrocyte cultures by glutamate, adenosine or elevated extracellular K^+^ concentrations, measured by a luciferin/luciferase reaction. It has also shown that this release is virtually abolished by an inhibitor of glycogenolysis as well as by inhibitors of transmitter-mediated signaling or of L-channel opening by elevated K^+^ concentrations.

## Abstract 2

Stimulation of Ca^2+^-dependent astrocytic ATP release by glutamate, adenosine or high K^+^ concentration is abolished by inhibition of glycogenolysis, probably due to signaling inhibition. This is consistent with the K^+^-mediated pathway, but glycogenolysis-sensitive steps in the transmitter pathways are unknown.

## INTRODUCTION

Stimulated release of ATP from astrocytes has repeatedly been shown. (i) Caciagli et al. (1988) found that electrical stimulation of cultured astrocytes pre-loaded with [^3^H]adenosine released approximately equal amounts of nucleosides and nucleotides to the medium in a Ca^2+^- and K^+^-dependent manner. (ii) Release of ATP from cultured astrocytes by glutamate, or by NMDA, AMPA or kainate-specific agonists was described by Queiroz et al. (1997). With addition of 100 μM glutamate they found an average ATP release of 0.3–0.4 pmol/min per mg of protein, but higher glutamate concentrations raised this value. (iii) Maienschein et al. (1999) confirmed release of ATP from cultured astrocytes by receptor-mediated mechanisms and suggested vesicular exocytosis as a potential mechanism of the release. These observations are important for astrocytic function, since one mechanism for the propagation of Ca^2+^ waves in astrocytes is by release of ATP (Leybaert and Sanderson, 2012), which subsequently activates purinergic receptors on adjacent cells (Cotrina et al., 2000). (iv) ATP release from cultured astrocytes was again found to be Ca^2+^-dependent by Bal-Price et al. (2002), who also described that a portion of astrocytic ATP is packaged in secretory granules. (v) Pangsric et al. (2007) showed punctate expression in cultured astrocytes of ATP-containing vesicles, which after glutamate stimulation fused with the plasma membrane in a Ca^2+^-dependent manner, leading to release of ATP. (vi) Pryazhnikov and Khiroug (2008) showed that exocytosis of ATP from cultured astrocytes was triggered by receptor stimulation or Ca^2+^ uncaging, but occurred after a delay, with only approximately 4% of the release during the first 30 s. (vii) In intact brain slices the time interval is even longer (Heinrich et al., 2012) suggesting activation of Ca^2+^-dependent signaling. (vii) Release of ATP from astrocytes gently isolated from brain slices (Lalo et al., 2006) has been demonstrated (Lalo et al., 2011). For studies of the role of glycogen in ATP release from brain slices or astrocytes obtained from brain slices it is important to remember that glycogen content may become drastically reduced when the animal is killed and only recover in the slices after at least 2 h of incubation (LeBaron, 1955).

Glycogenolysis is Ca^2+^-dependent, not only in muscle (Ozawa, 1972, 2011) but also in brain slices (Ververken et al., 1982) and leukocytes (Herlin et al., 1978). Glycogenolysis is an astrocyte-specific process (Gibbs et al., 2006), which is important for brain function both on account of its requirement for formation of transmitter glutamate (Gibbs et al., 2006) and because Na^+^,K^+^-ATPase-mediated K^+^ uptake in cultured astrocytes require glycogenolysis-dependent signaling (Xu et al., 2013). An intense K^+^ uptake triggered by extracellular K^+^ concentrations above 15 mM, which is mediated by activation of the Na^+^, K^+^, Cl^−^ and water transporter NKCC1, and is secondary to depolarization-mediated opening of L-channels for Ca^2+^ (Yan et al., 2013), also requires glycogenolysis. We therefore wondered whether K^+^-stimulated and perhaps also transmitter-stimulated ATP release might require glycogenolysis.

In the present paper this was studied by measuring ATP release from cultured astrocytes by a luciferin/luciferase method in the presence and absence of the glycogenolysis inhibitor 1,4-dideoxy-1,4-imino-D-arabinitol (DAB), with and without stimulation. In addition to testing the generally used but pathologically high K^+^ concentration of 45 mM we also tested whether a smaller effect could be obtained after addition of 10 mM K^+^, reaching a less pathological extracellular K^+^ concentration of 15 mM, and whether this effect might be increased by γ-aminobutyric acid (GABA) which is depolarizing in astrocytes and causes an increase in [Ca^2+^]_i_ (Bekar and Walz, 2002; Meier et al., 2008; Young et al., 2010; Ma et al., 2012; Egawa et al., 2013). GABA was also tested alone, as were the two other transmitters glutamate and adenosine.

## MATERIALS AND METHODS

### Reagents

Chemicals for preparation of medium and most other chemicals, including DAB, adenosine, glutamate, the glutamate receptor antagonist MPEP [2-methyl-6-(phenylethynyl)-pyridine], GABA and the ecto-ATPase inhibitor ARL67156 were purchased from Sigma. The inhibitor of MEK [MAPK (mitogen-activated protein kinase)/ERK (extracellular-signal-regulated kinase) kinase] U0126 was obtained from Calbiochem.

### Cell culture

Primary cultures of astrocytes were prepared from the neopallia of the cerebral hemispheres as previously described (Juurlink and Hertz, 1992) with minor modifications (Xu et al., 2013) and planted in 24-well plates in Dulbecco's Minimum Essential Medium (DMEM) with 7.5 mM glucose (to allow some decline between feedings) and the 5.4 mM K^+^ traditionally used in our cultures. After the age of 2 weeks, 0.25 mM dibutyryl cAMP (dBcAMP) was included in the medium. Such cultures are highly enriched in astrocytes (>95% purity of glial fibrillary protein- (GFAP-) and glutamine synthetase-expressing astrocytes (Hertz et al., 1985). The addition of dBcAMP at this specific stage of culturing is a crucial component of our culture preparation. It leads to a morphological and functional differentiation, as evidenced by the extension of cell processes, increases in several metabolic activities and expression of voltage sensitive L-channels for calcium (Ca^2+^), features which are characteristic of astrocytes *in situ* (Hertz, 1990; Meier et al., 1991, Yan et al., 2013). Use of astrocyte cultures has recently been authoritatively reviewed (Lange et al., 2012), and in our own cultures drug-induced changes in gene expression and editing have recently been confirmed in freshly isolated cells from mice treated with the same drugs (Li et al., 2012b; Song et al., 2012; Peng et al., 2013). Moreover the development of the glutamate/aspartate exchanger component aralar shows similar developmental patterns in the two preparations, with an increase in the cultured cells after dBcAMP administration (Li et al., 2012a).

### Luciferin/luciferase ATP assay

For determination of the extracellular ATP, a luciferin/luciferase assay (ENLITEN ATP Assay System Bioluminesecence Detection Kit, Promega) and a Tecan infinite M200 Microplate Analyzer were used to record relative light units (RLU) as described by Liu et al., (2008). Firefly luciferase catalyzes the oxidation of luciferin in the presence of ATP to produce light. For determination of ATP release the culture medium in 24-well plates was replaced with 180 μl of DMEM without pH indicator and serum but retaining its normal glucose content. To most of the incubation media an inhibitor of ecto-ATPase (ARL67156) was added at the beginning of the incubation. At the same time the glycogenolysis inhibitor (DAB) was added to some cultures. Fifteen minutes later a 60 min-incubation with drugs or excess K^+^ was begun by gently adding 20 μl of glutamate (1 mM in DMEM, to achieve a final concentration of 100 μM), of adenosine (1 mM in DMEM, to achieve a final concentration of 100 μM), of GABA (1 mM in DMEM, to achieve a final concentration of 100 μM), or of KCl (either 450 mM in DMEM to achieve a final K^+^ concentration of 50 mM or 100 mM in DMEM to achieve a final K^+^ concentration of 15 mM).

At the end of the 60-min experimental period a 100 μl aliquot of the incubation medium was quickly removed and its temperature reduced to 37°C for the ATP assay. It was mixed with a 100 μl aliquot of luciferin/luciferase reagent dissolved in dilution buffer provided with the assay kit, and chemiluminescence values (RLU) were recorded. Ionic salt sensitivity of the luciferase reaction was negligible, and it was not sensitive to DAB or other drugs employed in the present study (J. Xu, L. Hertz and L. Peng, unpublished work). However, even in the absence of any known ATP, considerable relative high RLU readings were caused by the medium and/or cultures (ATP is found in many compounds), and this varied slightly between experiments. A correction for RLUs that were independent of ATP release was introduced by adjusting the baseline of each figure, assuming that the relatively small ATP release under control conditions remained constant. This procedure ensures that relative changes are measured reasonably accurately. For calculation of absolute ATP levels, sample RLU readings were in some cases compared with RLU values obtained from standard solutions of ATP.

### Statistics

The statistical values of the differences between individual groups were analyzed by one-way ANOVA followed by Fisher's LSD test. The level of significance was set at *P*<0.05.

## RESULTS

### Effects of DAB

**Figure 1:**
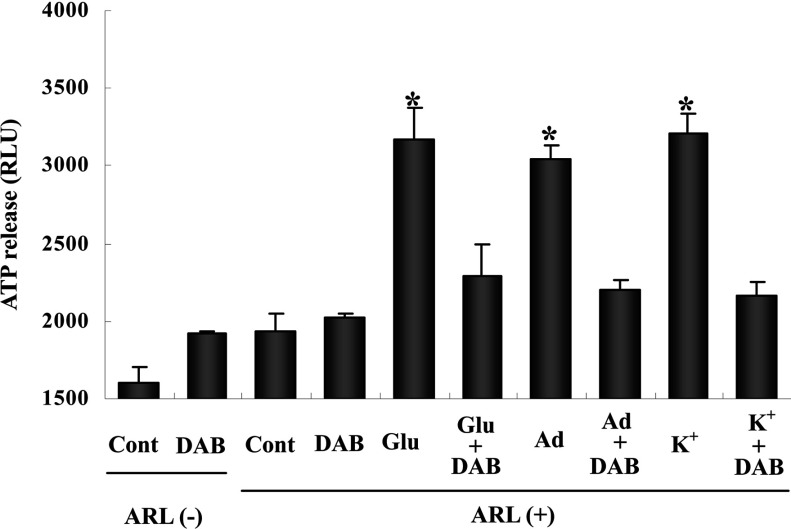
Glycogenolysis is required for the increase of ATP release from well differentiated astrocyte cultures induced by the addition of glutamate to a final concentration of 100 μM, adenosine to a final concentration of 100 μM or elevation of the extracellular K^+^ concentration by 45 mM The presence of any of these stimuli during incubation for 60 min in glucose-containing (7.5 mM) DMEM supplemented with the ecto-ATPase inhibitor ARL67156 increased the content of ATP, measured as RLU by a luciferin/luciferase technique. This increase was almost abolished in the additional presence of the glycogenolysis inhibitor (DAB). Results are the averages of RLU values from three to five individual cultures. S.E.M. values are indicated by vertical bars. *Statistically significant (*P*<0.05) difference from all other groups, but not from each other.

Without any tissue the incubation medium caused RLU values between 1200 and 1600 (J. Xu, L. Hertz and L. Peng, unpublished work) for the reasons discussed above. In Figure 1 all values are therefore indicated from a baseline of 1500. Under control conditions, without any ecto-ATPase inhibitor, there is virtually no visible increase in RLU (Figure 1). However, in the presence of the ecto-ATPase inhibitor ARL 67156 there is an increase of ~300 units, suggesting that a normally occurring small ATP release is obscured by ecto-ATPase action. An effect similar to that of ARL 67156 was caused by DAB, which might suggest that ecto-ATPase action requires glycogenolysis. This possibility was not further investigated, except by showing that the effects of ARL 67156 and DAB were not additive. In all remaining results of Figure 1 ARL 67156 was present. They illustrate the effects of 100 μM glutamate, 100 μM adenosine or an elevation of the extracellular K^+^ concentration by 45 mM (to a total of 50 mM). In the absence of DAB either agent increased RLU by up to 1000 RLU units, and this effect was almost completely inhibited by DAB.

**Figure 2:**
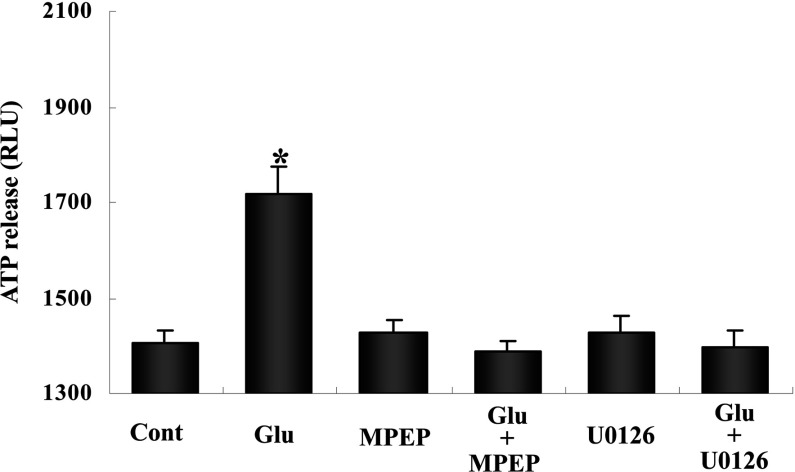
Glutamate-stimulated ATP release from astrocytes requires GluK2 activation and ERK1/2 phosphorylation The cells were treated as described in Figure 1 with the addition of glutamate to a final concentration of 100 μM glutamate, and the inhibitor of ecto-ATPase, ARL67156, present in all experiments. Some experiments were performed in the additional presence of either 25 μM MPEP, an antagonist of mGluR5 and GluK2 receptors, or 10 μM U0126, a specific inhibitor of ERK phosphorylation (by inhibition of MEK). For undetermined reasons the ATP release in response to glutamate was smaller than in Figure 1, but the main result is that it was completely prevented by either inhibitor. Literature data indicate that mGluR5 is not functioning in differentiated cells. Results are averages of RLU values from three individual cultures. S.E.M. values are indicated by vertical bars. *Statistically significant (*P*<0.05) difference from all other groups.

A standard curve (not shown) was used to estimate the corresponding amounts of ATP. It showed that 300 RLU units under control conditions corresponds ~0.3 nM and that a further increase of 1000 units under stimulated conditions equals ~5 nM. Since this value was obtained after a 1:1 dilution and the total volume of incubation was 200 μl, the total amount of ATP in the incubation medium must have increased by about 2 pmol. With a protein content of 35 μg per culture the amount of ATP released in response to glutamate during 60 min would thus amount to ~60 pmol or ~1.0 pmol/min per mg of protein. In the following two figures, ARL 67156 was present in all experiments to prevent ecto-ATPase activity. The ATP release shown in Figure 2 in response to glutamate was somewhat smaller than in Figure 1, perhaps reflecting differences in protein content in the cultures. However, it was still at least as high as the release found by Queiroz et al. (1997) in response to 100 μM glutamate. Addition of either the glutamate receptor antagonist MPEP or U0126 completely inhibited the glutamate-evoked increase in extracellular ATP. We have previously shown that MPEP inhibits glutamatergic signaling in astrocytes (Peng et al., 2012), and U0126 is a MEK inhibitor, which prevents phosphorylation of ERK1/2. ERK1/2 phosphorylation must accordingly constitute an important part of the glutamate-stimulated pathway leading to ATP release.

**Figure 3:**
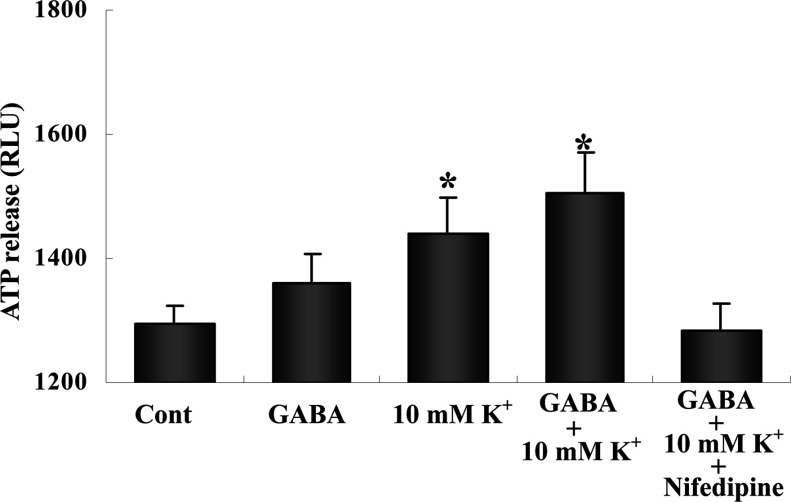
The cells were treated as described in Figure 2 but instead of adding glutamate the extracellular K^+^ concentration was increased by 10 mM, in some experiments together with the addition of GABA to a final concentration of 100 μM Either the addition of 10 mM K^+^ alone or the combined addition of K^+^ and GABA increased ATP release, although to a smaller degree than the higher, more depolarizing K^+^ concentration used in Figure 1. The response required L-Ca^2+^ channel activation, as indicated by its inhibition by 100 nM nifedipine, a dihydropyridine inhibitor of depolarization-opened L-channels for Ca^2+^ leading to increase in Ca^2+^. Results are averages of RLU values from three to five individual cultures. S.E.M. values are indicated by vertical bars. *Statistically significant (*P*<0.05) difference from control and GABA+10 mM K^+^+nifedipine.

Figure 3 shows that addition of 10 mM K^+^ caused a smaller release of ATP (about 300 RLU or ~0.3 pmol ATP/min per mg of protein) than exposure to higher K^+^ concentrations, and it made little difference whether or not GABA was also present. This release of ATP was abolished by nifedipine (Figure 3), a dihydropyridine inhibitor of L-channels for Ca^2+^, opened by depolarization and leading to an increase in free cytosolic Ca^2+^ concentration (Ca^2+^) (Yan et al., 2013). GABA alone seemed to cause a very small increase in ATP release, but it was far from being statistically significant.

## DISCUSSION

Abrogation of K^+^-stimulated release of ATP by inhibition of glycogenolysis could have been expected, since the L-channel-mediated pathway opened by the addition of 45 mM K^+^ (Cai et al., 2011) is inhibited when glycogenolysis is prevented (Xu et al., 2013). L-channel function is also known to be dependent on energy metabolism (Kostyuk, 1984). Elevation of extracellular K^+^ concentrations cause an increase in glycogenolysis both in intact brain tissue (Hof et al., 1988) and in cell cultures that have been treated with dBcAMP (Subbarao et al., 1995). The present study showed that also K^+^ addition resulting in a more physiologically relevant K^+^ concentration (10 mM) caused a significant release of ATP, which was completely inhibited by nifedipine. GABA alone had no significant effect and only slightly and non-significantly increased that of elevated extracellular K^+^ concentrations. The relatively small depolarization it causes seems insufficient to evoke ATP release.

The glycogenolysis-dependent pathways activated by glutamate and adenosine in astrocytes are unknown. The present study showed inhibition by the glutamate antagonist MPEP and by an ERK1/2 phosphorylation inhibitor. MPEP is generally known as an inhibitor of mGluR5, a metabotropic glutamate receptor, but this receptor is only functioning in astrocytes during very early development (Sun et al., 2013). MPEP appears also to inhibit the kainate receptor GluK2, present in mature astrocytes *in vivo* (Li et al., 2012a) and active in culture (Li et al., 2011). GluK2 is equally distributed in freshly isolated neurons and astrocytes (Li et al., 2012a). The signaling pathway for the GluK2 receptor in astrocytes has not been determined, but glutamate increases [Ca^2+^]_i_ in the presently used astrocytes (Li et al., 2011). However, in astrocytic transmitter pathways examined so far ERK1/2 phosphorylation is downstream of an increase in [Ca^2+^]_i_ and subsequent transactivation of the epidermal growth factor receptor (EGFR) (Peng, 2004; Peng et al., 2010). An inhibitor of ERK1/2 phosphorylation abolished glutamate-induced release of ATP, and we have evidence that inhibition of post-transamination signaling does not interfere with pre-transactivation signaling (J. Xu, L. Hertz and L. Peng, unpublished work). Accordingly, it can be concluded that increase in [Ca^2+^]_i_ is necessary, but not sufficient, for GluK2-mediated ATP release. Precise identification of the glycogenolysis-dependent step is therefore a major undertaking, beyond the scope of the present study.

Glutamate has been reported not to cause glycogenolysis in cultured astrocytes (Sorg and Magistretti, 1991). Consistent with this observation, 100 μM glutamate (the concentration used in this study) causes relatively little glycogenolysis also in our cultures, although it had a pronounced glycogenolytic effect at 1 mM (J. Xu, L. Hertz and L. Peng, unpublished work), perhaps due to massive glycogenolysis-requiring extrusion of K^+^ accumulated together with glutamate. In this context it is of interest that Queiroz et al. (1997) noted increased release of ATP when the glutamate concentration was raised beyond 100 μM.

Adenosine causes a stimulation of glycogenolysis in cultured astrocytes and brain slices, and at least in the cultured cells the stimulation is dependent upon cAMP signaling (Magistretti et al., 1986; Sorg and Magistretti, 1991), i.e. stimulation of an A_2_ receptor (van Calker et al., 1979). Both an A_2A_ and an A_2B_ receptor are now known to exist. A_2A_ adenosine receptors mediate their effects predominantly through coupling to adenylyl cyclase, but at least in some cells their activation might also induce formation of inositol phosphates, a rise in [Ca^2+^]_i_ and activation of protein kinase C (PKC) (Offermanns and Simon, 1995). This is important because cAMP as previously described can not induce glycogenolysis in the absence of an increase in [Ca^2+^]_i_ (Ozawa, 1972; 2011; Herlin et al., 1978; Ververken et al., 1982). However, Ca^2+^ can also enter the cell through receptors (Neary et al., 1988). Such a mechanism might perhaps explain why Kanno and Nisizaki (2012) found the release of glutamate and increase in [Ca^2+^]_i_ in cultured astrocytes in response to adenosine to require protein kinase A function but not Ca^2+^ release from either Ins*P*_3_ or ryanodine receptors. Whether this applies also for the present release of ATP is unknown.
